# Rawls and Animal Moral Personality

**DOI:** 10.3390/ani13071238

**Published:** 2023-04-03

**Authors:** Guy Baldwin

**Affiliations:** Faculty of Law, University of Cambridge, 10 West Road, Cambridge CB3 9DZ, UK; gjb61@cam.ac.uk

**Keywords:** animal rights, John Rawls, moral personality, moral powers, moral status, conception of the good, sense of justice, theory of justice

## Abstract

**Simple Summary:**

“Moral personality” is required in order to be entitled to justice in John Rawls’s theory of justice, a famous and influential theory in political philosophy. The concept of moral personality involves the possession of two “moral powers”. One moral power is a capacity for a conception of the good, being a conception of what is regarded as worthwhile in life, while the other is a capacity for a sense of justice. Rawls claims that non-human animals (hereafter, “animals”) do not possess these moral powers, and accordingly he omits them altogether from his theory of justice. In this article, I raise doubts about this omission, outlining how at least some animals may indeed possess the moral powers, albeit to a lesser extent than most humans. In this regard, the distinction between humans and animals can be seen as one of degree rather than kind. A proper acknowledgement of animal abilities suggests that Rawls’s theory requires alteration to accommodate the position of animals.

**Abstract:**

The relationship between animal rights and contractarian theories of justice such as that of Rawls has long been vexed. In this article, I contribute to the debate over the possibility of inclusion of animals in Rawls’s theory of justice by critiquing the rationale he gives for their omission: that they do not possess moral personality. Contrary to Rawls’s assumptions, it appears that some animals may possess the moral powers that comprise moral personality, albeit to a lesser extent than most humans. Some animals can act in pursuit of preferences and desires (and communicate them non-verbally), which might be taken as implicitly selecting a conception of the good; further, scientific research demonstrating inequity aversion and social play behaviors suggests that some animals can have a sense of justice relating to their own social groups. I conclude that Rawls’s theory needs to acknowledge any animals that can be considered to meet the threshold of moral personality, while the concept of moral personality as a range property may also require reconsideration.

## 1. Introduction

A limitation of John Rawls’s theory of justice is that the position of non-human animals (hereafter, “animals”) is treated as outside the scope of the theory. Although Rawls considered cruelty towards animals and the destruction of an entire species to be wrong, animals are said not to be entitled to justice [1] (pp. 441–442, 448–449). The reason offered for this exclusion is that animals are not “moral persons”. Moral personality involves two moral powers—the capacity for a conception of the good and the capacity for a sense of justice—that are claimed to be uniquely human attributes. Rawls’s position seems to mean that, as Martha Nussbaum puts it, “[e]ven … the twentieth century’s greatest philosopher of justice … held that it was virtuous to treat animals with compassion, but that they could not be treated justly or unjustly” [2] (pp. 8–9).

The relationship between animal rights and contractarian theories of justice such as that of Rawls has long been vexed; a key difficulty is how to include animals in such theories if they are unable to participate effectively in the making of a social contract [3]. Mark Rowlands argues that “there is nothing in contractarianism per se that requires the contract be restricted to rational agents”; even if the framers of the contract have to be rational agents, its recipients do not (p. 236). Meanwhile, Robert Garner claims that Rawls’s approach cannot adequately explain the position of “those humans who are less endowed with rationality or autonomy”, invoking the well-known argument from so-called “marginal cases” [4] (p. 7), though he has also critiqued this argument [5]. The contribution of this article is to consider a different, under-explored issue: the merits of Rawls’s claim that animals do not have moral personality, which underpins their exclusion from his theory of justice.

In ascribing moral personality only to humans, Rawls treats humans and animals as qualitatively different. Rawls’s concept of moral personality is widely assumed to “clearly preclude animals” [6] (pp. 2–3). However, as Rowlands says, “the all or nothing manner in which discussions of non-human rationality tend to be discussed is eminently questionable, on both theoretical and methodological grounds” (p. 236). Indeed, I argue that some animals might be better viewed as possessing moral personality, though to a more limited extent than most humans. The difference between humans and animals in this regard may be thought of as one of degree rather than kind, to adopt Charles Darwin’s words [7] (p. 179). Nonetheless, acknowledgment of the lesser degree of moral personality possessed by some animals is likely to require significant alterations to Rawls’s theory. Although I seek to establish some problems with Rawls’s account, modifying the theory to accommodate the possibility of animal moral personality is not attempted here. Further, I do not suggest that possession of some measure of moral personality means that animals (or, for that matter, humans) cannot or do not perform acts that inflict suffering on others.

The article proceeds in three parts. First, I address Rawls’s analysis of the position of animals in his theory. As will be shown, Rawls’s theory is already consistent with the view that animals are owed moral concern. Nothing prevents legislators from protecting animal welfare or rights. However, Rawls considers that due to their claimed absence of moral personality, animals are not owed justice, and this may mean that animal protection is of lower priority than the principles of justice. Second, I question Rawls’s approach by analyzing the position of animals in respect of each of the moral powers. I suggest that Rawls’s conclusion that animals do not have moral personality is too simple; there is reason to consider that at least some animals can potentially possess the moral powers, albeit to a lesser degree than humans. Third, I conclude with a short reflection on the possible implications of this analysis for Rawls’s theory.

## 2. Rawls and Animals

In *A Theory of Justice*, Rawls only briefly addresses the position of animals. He describes “the basis of equality” as “the features of human beings in virtue of which they are to be treated in accordance with the principles of justice” and claims that “[o]ur conduct toward animals is not regulated by these principles, or so it is generally believed” (p. 441). That is because it is the “moral persons” who are entitled to equal justice. Such moral persons are “capable of having (and are assumed to have) a conception of their good (as expressed by a rational plan of life)” and “capable of having (and are assumed to acquire) a sense of justice, a normally effective desire to apply and to act upon the principles of justice, at least to a certain minimum degree”. Thus, “equal justice is owed to those who have the capacity to take in and to act in accordance with the public understanding of the initial situation” (p. 442).

Moral personality refers to a capacity, not its realization; a being that has the required capacity, whether or not it is developed, receives “full protection” (pp. 445–446). Moreover, it is a “range property”, similar to how “the property of being in the interior of the unit circle is a range property of points in the plane”, because all points inside the circle have the property of being in the interior and they have the property equally (p. 444). In the same way, “provided the minimum for moral personality is satisfied, a person is owed all the guarantees of justice” (p. 443). However, Rawls considers that animals do not meet this minimum, and although Rawls describes moral personality as a sufficient condition for being entitled to equal justice and leaves aside whether it is also a necessary condition (pp. 442–443), he seems to treat it as a necessary condition in the case of animals. He explains his view of the position of animals in a key passage:

Last of all, we should recall here the limits of a theory of justice. Not only are many aspects of morality left aside, but no account is given of right conduct in regard to animals and the rest of nature. A conception of justice is but one part of a moral view. While I have not maintained that the capacity for a sense of justice is necessary in order to be owed the duties of justice, it does seem that we are not required to give strict justice anyway to creatures lacking this capacity. But it does not follow that there are no requirements at all in regard to them, nor in our relations with the natural order. Certainly it is wrong to be cruel to animals and the destruction of a whole species can be a great evil. The capacity for feelings of pleasure and pain and for the forms of life of which animals are capable clearly imposes duties of compassion and humanity in their case. I shall not attempt to explain these considered beliefs. They are outside the scope of the theory of justice, and it does not seem possible to extend the contract doctrine so as to include them in a natural way.

(p. 448)

In short, then, Rawls does not accept that animals are capable of a sense of justice, and therefore we are not required to give “strict” justice to creatures lacking this capacity. However, Rawls’s position that animals are not entitled to “strict” justice raises questions. How do we square this with his view that cruelty towards animals or the destruction of an entire species would be wrong? Presumably, these may be moral duties, but not duties of justice or at least not of “strict” justice. This position is open, of course, to objection that animals might conceivably be owed justice on the basis of their sentience or consciousness [8,9], or experience of pain and pleasure [10], without it being necessary to demonstrate that they themselves have a sense of justice. For Rawls, it seems that such matters might suffice to demonstrate that animals are due moral consideration, but not justice (or “strict” justice).

In other words, as Ruth Abbey puts it, Rawls “makes it clear that humans have duties to animals that derive not from the considerations of justice, but from those of morality” (p. 6). Rawls’s position therefore does not seem to preclude animal welfare laws or even animals holding non-basic liberties—legislators can presumably grant animals welfare protections or rights even if this is not compelled by the principles of justice, but merely considerations of morality. Indeed, animal welfare laws have been introduced in many jurisdictions. From one perspective, animals may already have “simple” rights under these welfare laws [11]. Legislators might even choose to confer “fundamental” rights on animals. Such conferrals of rights may take place on the basis of the sentience of animals or other considerations.

One might then query the relevance of the analysis in this article. Surely, it might be said, it is *human* moral personality that matters, as humans make use of their moral reasoning to appreciate their duties to animals. However, Rawls’s position has potentially adverse consequences for animals, including that animal interests might be subordinated to the human interests protected by the principles of justice [12]. A particular difficulty is that the interests of animals could seemingly be overridden by human basic liberties protected by the first principle of justice (the liberty principle). Pursuant to that principle, Rawls outlines a list of basic liberties required for the “adequate development and full exercise of the two moral powers” [13] (p. 45). Since only humans have these basic liberties, any rights of animals appear to be in a lesser position, no matter the relative importance of the interests involved.

For example, religious practices are protected by liberty of conscience (often referred to as religious freedom), which is one of the basic liberties within the first principle of justice. As such, if animal sacrifice is a religious practice protected by a basic liberty adopted as part of the basic structure of society, it might be considered to take priority over animal protection laws. According to Rawls’s explanation in *A Theory of Justice*, basic liberties can be limited when they conflict with other basic liberties, particularly outside what he calls their central range of application (p. 54). He also acknowledges possible limitations on basic liberties in the common interest in public order and security (pp. 186–189). Whether animal protection laws would be subject to the exercise of a basic liberty may therefore depend on whether those laws validly limit a basic liberty. Nonetheless, the failure to acknowledge animal basic liberties creates a risk that a human basic liberty will take precedence over animal interests.

This possibility is not merely hypothetical. The case of *Lukumi Babalu Aye v City of Hialeah* [14], in which the US Supreme Court struck down laws targeting animal sacrifice by Santeria practitioners on the basis that they violated the Free Exercise Clause of the First Amendment to the US Constitution, serves as an illustration of the possibility of prioritization of human rights over even the lives of animals. However, when the interests protected by a human basic liberty do not seem as important as, say, the life of an animal, that may clash with an intuition that “the basic or fundamental interests of animals should not be sacrificed for the sake of the trivial or non-basic interests of humans”, as Garner puts it (p. 11). The question that arises is whether animals can be moral persons whose fundamental interests can be protected by principles of justice at the same level as human interests.

In analyzing moral personality, I adopt a different focus from other recent accounts of animals that draw on Rawlsian ideas. For example, Federico Zuolo employs the concept of public justification to develop a theory that builds on the presence of reasonable disagreement about animals [15]. Laura Valentini argues that, from the perspective of an associative account, justice requires that the interests of domesticated dogs “be taken into account in the design of laws and policies within our political communities” [16] (p. 49). That is because, given the history of cooperation between humans and dogs, they can be considered to be fellow cooperators that are objects of moral concern (p. 38). Although these approaches are promising, I do not address public justification or animal cooperation with humans, instead offering a critique of Rawls’s assumptions about animal abilities.

## 3. Animal Moral Personality?

Are the moral powers that constitute moral personality exclusive to human beings? If they are not, then we may question Rawls’s conclusion that animals are not entitled to strict justice and his consequent omission of them from his theory of justice. As Raffael Fasel points out, “[i]dentifying the precise features of human nature that made humans (and only humans) special” has long “proved difficult” [17] (p. 4). In this section, I consider each of the moral powers—the capacity for a conception of the good and the capacity for a sense of justice—and conclude that some animals can potentially possess the moral powers, albeit to a more limited extent than humans. In part, the analysis reflects the fact that our scientific understanding of animal behavior and cognition has progressed since Rawls wrote *A Theory of Justice*. However, since we still have an incomplete understanding of animal abilities, the analysis remains subject to possible future changes in scientific understanding.

One difficulty is that moral personality is a range property, such that those who fall short are considered not to have moral personality at all, while those who meet the threshold to any degree have full moral personality [18,19]. There is reason to consider that some animals may well qualify as moral persons even though their extent of moral personality is less than most humans; many other animals are likely to fall short. To adapt Rawls’s theory in a way that makes it truly non-anthropocentric, it might be necessary to rethink the understanding of moral personality as a range property, though whether moral personality could be reconceived as scalar or as having gradations that reflect lesser degrees of moral personality—which would constitute a significant alteration to the theory—is a complex question. In this analysis, I consider moral personality as a range property, though I note the possibility of alternative approaches in the conclusion.

A further difficulty that would arise in an attempt to adapt Rawls’s theory to include animals is determining which animals reach the threshold of moral personality. Marc Bekoff and Jessica Pierce, commenting on the possibility of identifying animals that constitute “moral animals” in the sense in which they use the term, observe that “[g]iven the rapidly accumulating data on the social behavior of numerous and diverse species, drawing such a line is surely an exercise in futility, and the best we can offer is that if you choose to draw a line, use a pencil” [20] (p. 8). However, they outline a preliminary list of primates, social carnivores, cetaceans and some rodents for which they consider there is “compelling evidence for moral behavior” (p. 9). The same kind of evidentiary limitations apply to the question of which animals might qualify as Rawlsian moral persons based on our current knowledge; the answer may shift over time.

In raising the possibility of animal moral personality, I do not seek to rely on the so-called “marginal cases”—humans who are too severely disabled or unwell to have the capacity for moral personality. Rawls emphasizes that what is required for moral personality is the *potential* to exercise and develop the moral powers “that is ordinarily realized in due course” (p. 442). Thus, infants and children are included, as are those who have lost their capacity temporarily. However, it could be objected that Rawls does not account for the moral status of those humans whose incapacity may be permanent or lifelong. He observes that the problem of “those more or less permanently deprived of moral personality may present a difficulty” but declines to examine it (p. 446). One possible approach may be to accept that some “scattered” humans, as Rawls puts it (p. 443), do not meet the threshold. In respect of animals, though, the problem is different: it is that Rawls overlooks the actual abilities of many animals, which go a significant way towards human moral personality. Thus, it is to the issue of the abilities of animals that I turn.

### 3.1. A Conception of the Good

In *A Theory of Justice*, Rawls explains the moral power of a capacity for a conception of the good as involving a “rational plan of life” (p. 442). In *Political Liberalism*, the moral power is defined as “the capacity to form, to revise, and rationally to pursue” a conception of the good, being “a conception of what we regard for us as a worthwhile human life”, which normally consists of a “determinate scheme of final ends and aims, and of desires that certain persons and associations, as objects of attachments and loyalties, should flourish” [21] (p. 302). In *Justice as Fairness: A Restatement*, Rawls describes such a conception as “an ordered family of final ends and aims which specifies a person’s conception of what is of value in human life or, alternatively, of what is regarded as a fully worthwhile life”; he adds that the elements of such a conception are “normally set within, and interpreted by, certain comprehensive religious, philosophical, or moral doctrines” (pp. 18–19).

Setting aside the reference to “human” life, which may be viewed as question begging for present purposes, one initial difficulty is that animals usually cannot speak to us to articulate a conception of the good (subject to the possibility of, for example, a primate learning sign language, or other similar developments). Nonetheless, some animals can (and do) make choices as to their preferences. This is similar to what Tom Regan calls “preference autonomy”—that is, “the ability to initiate action because one has those desires or goals one has and believes, rightly or wrongly, that one’s desires or purposes will be satisfied or achieved by acting in a certain way” [22] (pp. 84–85). Moreover, there are animals that can signal their desires to us through other, non-verbal means. The question is whether acting in pursuit of certain stable preferences (and communicating them non-verbally) might, on one view, be taken as implicitly selecting a “conception of what is of value” or “worthwhile” in that animal’s life—in other words, an animal’s conception of the good.

Consider, by way of illustration of this, a simple hypothetical example that may be familiar to any owner of a companion animal [23]: a domesticated dog that chooses a favorite chair to sit in in the house when his owner is away. Why he chooses this chair may not be altogether clear: maybe because it is in the sun, or because it is close to the food bowl, or for any other conceivable reason—perhaps because he just likes the chair. Nonetheless, he consistently chooses this chair. On one view, this preference reflects the dog’s “conception of what is of value” or “worthwhile” in his life. That is to say, he is making a choice as to what he prefers in a place to sit and thus what the dog is implicitly doing is to form and pursue a conception of what is of value or worthwhile in his life.

Can a dog also “revise” his conception of the good, as required by the other verb Rawls uses? It is hard to see why not. The dog is certainly able to change his mind about his favorite chair. Perhaps, after a long time favoring a particular chair, he simply grows tired of it and decides to sit elsewhere in future. Or perhaps he has come to realize, walking on the way to his favorite chair, that a sofa in a different room gets the afternoon sun in a particularly appealing way, and that becomes his new favorite. In so doing, the dog revises his conception of the good. Moreover, although the example given is of a domesticated dog, the argument may apply equally to wild animals if they have the required abilities.

However, there are at least three possible challenges to this view, being ways in which such behavior by animals—making choices as to their preferences—might be considered not to fully match Rawls’s description of this moral power. The first issue that could be raised concerns the immediacy of the aims being pursued. In defining this moral power, Rawls refers to having a “plan of life” or a “determinate scheme of final ends and aims”. In the case of animals, the conception of the good in question may not involve a “plan of life” in a long-term sense, or constitute a “determinate scheme of *final* ends and aims”—as far as we know, at least, the ends and aims are often shorter term, or in the case of some activities involving planning, medium term, rather than “final”.

Nonetheless, it seems clear that animals can plan: consider the case of a bird building a nest for her young, or migrating to some distant place, or a squirrel burying nuts to eat later, or other examples of future planning documented in the scientific literature on “mental time travel” [24,25]. Such behavior appears to reflect ends and aims that, although not “final”, may sometimes lie rather distantly in the future. As Regan says, animals can be “individuals with desires, beliefs, and the ability to act in pursuit of their goals” (p. 116). Those goals are not always immediate and may be medium term or even somewhat long term. Nor is it the case that humans are constantly contemplating their “final” ends (we might also ask whether our ends and aims are ever meant to be truly final, until they turn out to be). Moreover, humans routinely engage in actions that are motivated by immediate or short-term desires; for many of us, long term planning is not a strong point. This does not deny humans moral personality, and nor should it for animals.

The second issue is whether animals can have “desires that certain persons and associations, as objects of attachments and loyalties … should flourish”. The concern shown by a domesticated dog for an injured or unwell owner seems to suggest that this is possible [26]. Again, the dog might not have as developed a sense of the human’s life—and would not be thinking, for example, about the human’s job prospects or other aspects of a flourishing life that lie beyond the dog’s comprehension of human civilization—but it seems wrong to suggest that the dog lacks the desire for his owner, as an object of attachment and loyalty, to flourish. Once again, the limitations of the abilities of animals do not seem to deny their possession of moral personality entirely, but merely to constrain its extent.

The third issue is whether animals are forming, revising and pursuing their conception of the good “rationally”, in the sense of a “rational” plan of life. Rawls explains that a rational plan of life involves a rational choice, and awareness of the relevant facts and consideration of the consequences (pp. 358–359). This may appear to be a point of difficulty for the argument. On the one hand, there is obviously a thought process behind animal choices, which may include consideration of the circumstances and the possible consequences of actions. Although it can be accepted that an animal is not espousing a comprehensive doctrine on one’s relation to the world, Rawls stipulates that this is “normally” an aspect of a conception of the good, a necessary qualification since many humans might not espouse any comprehensive doctrine; it does not seem required to demonstrate rationality. Rowlands observes that “the claim that animals are not rational is plainly false on at least some conceptions of rationality. Animals can identify causal relations and use their understanding of such relations to solve problems” [27] (pp. 78–79).

On the other hand, a concern may be raised that animals are not reflective enough. As part of an argument that most animals do not have an interest in liberty, Alasdair Cochrane observes that “we are on reasonably safe ground to attribute to sentient animals the ability to possess and pursue desires” [28] (p. 667). However, seeking to reject the “autonomy” of particular animals, he claims that “[m]ost animals cannot frame, revise, and pursue their own conceptions of the good. This is not to say that sentient animals do not have different characters, nor is it to deny that they can make choices. It is simply to make the point that most animals cannot forge their own life plans and goals” (p. 668). As to why “most” animals are unable to do this, Cochrane relies on their (presumed) lack of reflective capabilities. He says that what is required is “the capacity to reflect on … desires, and modify them in relation to one’s own conception of the good” (p. 667).

According to Cochrane, “such a capacity requires a level of consciousness above mere sentience. Indeed, one might claim that to be an autonomous agent one needs to possess ‘higher-order thought consciousness’, that is, to be able to have thoughts about thoughts” (p. 667). However, there are two difficulties with employing such a requirement as the basis for excluding animals from Rawls’s moral power of a capacity for a conception of the good. First, it seems to add to the list of verbs—what is required, on this approach, is not merely forming, revising and pursuing a conception of the good, but also reflecting upon it, or upon other desires in relation to it. It seems conceivable for animals to effectively pursue a conception of the good, embodying a set of stable preferences, without necessarily reflecting on other desires from the standpoint of that conception—simply by faithfully adhering to those stable preferences until they are revised.

Second, although the scientific literature shows evidence of animal metacognition [29], and even deception [30], at this stage we do not know how reflective animals truly can be, including in relation to their desires. Given that some animals have stable preferences, it might be surprising if they were unable to assess other desires by reference to those preferences. A dog that chooses to sit in his favorite chair may well be considering and dismissing other impulses that pop into his head on the way there. However, we cannot say with any confidence how much reflection is taking place. That cuts both ways: a *lack* of reflectiveness is also not a sound basis on which to exclude animals from moral personality, because it is based on unproven assumptions about their abilities. Until scientific research clarifies the extent of animal metacognition, this will remain shaky ground for possible opposition to animals’ capacity for a conception of the good.

In short, although it may be accepted that there are some limitations on animals’ capacity for a conception of the good, those limitations do not detract from the point that they often seem capable of forming, revising, and pursuing a conception of what is of value and worthwhile in their lives, depending, of course, on the abilities of the individual animal or species, but recalling that what is required for moral personality is merely a capacity, not its realization. In the case of animals, this capacity clearly does not rise to the level of sophistication of a human capacity for a conception of the good, and it perhaps may not extend as far into the future, but the difference might be viewed as one of degree, rather than of kind. The behavior of animals constitutes evidence that, rather than not possessing the moral power of a capacity for conception of the good at all, many animals might indeed possess it, albeit in a more limited sense than most humans do.

### 3.2. A Sense of Justice

In *A Theory of Justice*, Rawls defines the moral power of a capacity for a sense of justice as “a normally effective desire to apply and to act upon the principles of justice, at least to a certain minimum degree” (p. 442). In *Political Liberalism*, he refers to it as “the capacity to understand, to apply, and normally to be moved by an effective desire to act from (and not merely in accordance with) the principles of justice as the fair terms of social cooperation” (p. 302). In *Justice as Fairness: A Restatement*, he describes it in similar terms as “the capacity to understand, to apply, and to act from (and not merely in accordance with) the principles of political justice that specify the fair terms of social cooperation” (pp. 18–19). Any argument around animals’ sense of justice rests on incomplete scientific knowledge of their behavior and cognition, as well as difficult philosophical questions about the meaning of “justice” and a “certain minimum degree”. However, there is reason to doubt Rawls’s assumption that animals necessarily cannot have a sense of justice.

It can be acknowledged at the outset that animals may not have a sense of justice in the sense of understanding, applying and acting from Rawls’s own principles of justice, which relate to the basic structure of a human society. These principles, particularly the difference principle, might not be comprehensible to many humans; we cannot suppose that there are any animals capable of understanding them. However, it seems problematic to say that animals therefore cannot have any sense of justice, because even if they cannot understand, apply and act from the principles of justice for a human society that are formulated by Rawls in his theory of justice, there is scientific evidence that suggests that some animals may have a sense of justice relating to their own social groups.

Drawing on scientific research, Bekoff and Pierce argue that animals do have a sense of justice in this way (p. 113). They define justice as

a set of expectations about what one deserves and how one ought to be treated. Justice has been served when these expectations have been appropriately met. Our justice cluster comprises several behaviors related to fairness, including a desire for equity and a desire … and a capacity to share reciprocally. The cluster also includes various behavioral reactions to injustice, including retribution, indignation, and forgiveness, as well as reactions to justice such as pleasure, gratitude, and trust.

Contending that animals can be “moral subjects” that can act for moral reasons, but not “moral agents” that are morally responsible for their actions (issues not addressed in this article), Rowlands objects to the definition of justice offered by Bekoff and Pierce. He writes that “a ‘set of expectations about what one deserves and how one ought to be treated’ does not add up to justice—at least as this is ordinarily understood—for the simple reason that one can have unjust expectations about what one deserves or how one ought to be treated” (p. 30). He goes on to say:

If we identify justice with any behavioral norm that happens to be in place in a social group, then who would wish to deny that animals have a sense of justice? That is, who would wish to deny that there are norms of behavior that regulate the interactions between members of a social group, and that an individual’s violation of those norms can often lead to conflict? Without such norms and their enforcement, it is difficult to see how any social group could remain in existence.

(p. 32)

However, there is a certain imprecision to the way in which Rowlands characterizes Bekoff and Pierce’s definition. According to Bekoff and Pierce, it is not any behavioral norm within a social group that constitutes justice; it is one relating to what one deserves and how one ought to be treated, a more specific subset of norms. Rowlands also objects that one may have “unjust expectations”. However, that seems to amount to merely saying that one’s sense of justice may be incorrect. The issue is perhaps better expressed not as whether one’s expectations are unjust or not—humans’ sense of justice may frequently be wrong—but whether the expectations relate to a concept of fairness (whether correct or not). Although this element of fairness is omitted from Bekoff and Pierce’s definition, they refer to fairness on many occasions elsewhere, so they too seem to be aware of its importance.

There is a significant amount of scientific evidence that animals can have such expectations of treatment relating to fairness, and understand, apply and act from them. One famous example is a study in which Sarah Brosnan and Frans de Waal demonstrated inequity aversion in capuchin monkeys through an experiment in which pairs of monkeys exchanged tokens for rewards, and one was given a cucumber and the other a grape (a preferred reward) [31]. Brosnan and de Waal summarized their results as that “a nonhuman primate, the brown capuchin monkey … responds negatively to unequal reward distribution in exchanges with a human experimenter. Monkeys refused to participate if they witnessed a conspecific obtain a more attractive reward for equal effort, an effect amplified if the partner received such a reward without any effort at all” (p. 297). The capuchin monkeys appeared to respond negatively to a situation that they perceived as unfair, even forgoing their own reward due to the perceived unfairness. Similar results have been found in some other animals, such as chimpanzees [32].

An additional example is social play behavior, which Bekoff and Pierce consider to offer “the most compelling evidence for a sense of fairness in social mammals”, both wild and domesticated (p. 115). They point out that some animals, such as dogs, coyotes, and wolves, “exhibit fairness during play, and they react negatively to unfair play behavior. In this context, fairness has to do with an individual’s specific social expectations, and not some universally defined standard of right and wrong” (p. 120). Unfair play can include behaving aggressively or attempting to mate. Thus, “during play, when a dog becomes too assertive, too aggressive, or tries to mate … the violation of trust stops play, and play only continues if the playmate ‘apologizes’ by indicating through gestures such as a play bow his intention to keep playing” (p. 121). Play, too, can be seen as an instance of animals understanding, applying and acting from a set of expectations—by enforcing them against other participants—about what they deserve and how they should be treated in a way that relates to fairness.

The scientific literature is also replete with examples of animal altruism [33], and there is evidence for animal empathy as well [34,35,36]. Is this relevant to a sense of justice? The presence of altruistic behavior does not necessarily demonstrate a sense of justice, or at least not directly, because one can assist another without considering what that other should be able to expect in terms of what is fair. Nonetheless, altruism helps to illustrate the forms of social cooperation that exist among animals, to which inequity aversion and social play behaviors may be closely related.

However, there are at least two possible challenges to the foregoing analysis of an animal sense of justice. The first—a possible response to any argument for animal morality, including one that claims that some animals can have a sense of justice—is that the behavior in question merely reflects instinct. However, to the extent that that is so, it does not seem to be a real point of differentiation between animals and humans, since in both cases moral behavior is the result of a combination of learned practices and instinct resulting from evolution. As Nussbaum explains:

No doubt [animal] behavior has its basis in instinct, but here we should make two points. First, our own moral behavior is also based on our instinctual evolutionary endowment: an inherited tendency to assist others has helped humans to survive and flourish. Second, both humans and other animals need teaching to develop their instincts in an appropriate way. We see this cultural element in animal behavior clearly when we live with animals: dogs who are not appropriately trained behave lawlessly, and can even be schooled into dangerous aggression (as when pit bulls, who can be loving and cooperative, are trained, instead, to attack). Dogs who are well-trained internalize norms pertinent to their conduct.

(p. 74)

Indeed, discussing Rawls’s psychological account of how a sense of justice is acquired by humans (as set out in chapter VIII of *A Theory of Justice*), Robert Elliot points out that there are overt parallels between the process by which they “begin to understand their place in an association and to appreciate how they and others may benefit from co-operative, regulated behaviour” and cooperation in animal societies, such as a wolf pack [37] (p. 99). He adds:

Rawls is quite prepared to support his principles of justice in a way that echoes ethologists’ explanations of animal behaviour. He says that “the capacity for a sense of justice and the moral feelings is an adaption of mankind to its place in nature” and elaborates, saying that, “for members of a species which lives in stable social groups, the ability to comply with fair co-operative arrangements and to develop the sentiments necessary to support them is highly advantageous, especially when individuals have a long life and are dependent on one another”.

(p. 100)

The second possible objection is that the expectations of treatment relating to fairness that some animals may apply and act upon do not correspond with the principles of justice as the fair terms of social cooperation that Rawls is invoking in his definition of this moral power. That objection has salience if the focus is on *human* principles of justice, including the principles of justice adopted in respect of human society’s basic structure in Rawls’s theory. As acknowledged above, we have no reason to consider that animals can understand, apply and act from these principles. However, what the evidence seems to suggest is that some animals may understand, apply and act from what might be described as principles that specify fair terms of social cooperation governing the workings of their own social groups.

For example, monkeys might not be able to consider the justice of a human political institution, but monkeys can have societies of their own, albeit at a much smaller scale, whose justice they may consider when they take issue with the fairness of others members’ behavior. Further, as the inequity aversion study suggests, they also seem able to take issue with the fairness of human behavior towards them. Thus, although it may be accepted that animals, in applying their own sense of justice, would not be “tak[ing] part in and … act[ing] in accordance with the public understanding of the initial situation” in a Rawlsian sense (p. 442), the fact that animals can have their own societies suggests that their failure to consider the justice of *human* society may be beside the point in establishing their abilities in respect of this moral power.

To the extent that some animals can have a set of expectations about treatment relating to fairness, they seem able to understand, apply and act from their own sense of justice, albeit to a lesser degree and at a smaller social scale than most humans. Nussbaum observes that rather than a “sharp binary division” between the moral rationality of humans and animals, there is really “a continuum”, reflecting a shared evolutionary history between our different species (p. 73). Indeed, humans can be said to be on a different point on a continuum from those animals that possess a sense of justice to a more limited extent. As with animals’ capacity for a conception of the good, Rawls’s claim that animals lack any capacity for a sense of justice appears to be, at best, a misleading characterization of a more nuanced and complex reality.

That is not to suggest that recognition of an animal sense of justice would be a simple matter for Rawls’s theory; there is no doubt that it raises difficulties. One particular difficulty is that, even if some animals are accepted as having a sense of justice relating to their own social groups, so long as those animals do not have a sense of justice relating to a human society, they may be unable to engage with humans in a reciprocal way consistent with Rawls’s principles of justice as the fair terms of social cooperation. What this indicates is that, in order to be appropriately inclusive of animals and their abilities, a significant reconsideration of aspects of Rawls’s theory may be required. However, such a reconsideration seems to be unavoidable if the theory cannot convincingly explain the basis for the exclusion of animals from considerations of justice.

## 4. Conclusions

Although Rawls does not dispute that animals merit moral consideration, they are omitted from his theory of justice. The basis for this omission is the purported absence of animal moral personality. However, this omission seems doubtful. Some animals may have, to a certain extent, capacities for a conception of the good and a sense of justice. The difference in this regard between humans and animals may be better viewed as one of degree, not kind. Consequently, a deficiency of Rawls’s approach is that it offers no recognition of the abilities of animals. As Garner says, “it seems entirely appropriate to include the interests of animals within the justice equation. To do otherwise implies a qualitative difference between humans and animals that the evidence suggests does not exist” (p. 13).

In part, that reflects advancements in our understanding of the behavior and cognition of animals that had not occurred yet when Rawls originally published *A Theory of Justice* in 1971. It is possible that with the benefit of those advancements in understanding, Rawls would have had a different, more nuanced view about the position of animals within his theory, on the basis that it is too simplistic to say that animals as a class do not possess moral personality. However, it is also possible that he would have maintained the position that it is legitimate to confine the scope of a theory of justice to humans. Nonetheless, the limitation in scope to humans diminishes the persuasiveness of his theory if it is justified in a way that overlooks the actual abilities of animals.

There are two possible approaches to addressing this exclusion of animals from Rawls’s theory. The first possibility is that, if it is accepted that some animals meet the threshold of moral personality, then those animals are acknowledged as moral persons entitled to justice. However, many animals might not be thought to meet the threshold. The second possibility is to reconsider moral personality, either to treat it as scalar or to recognize a lesser level of moral personality that encompasses the abilities of a larger group of animals. The problem is that if moral personality is considered scalar, then humans might not possess it equally among themselves. Alternatively, if a lesser level of moral personality is recognized, then it would be necessary to determine the scope and implications of this form of moral personality. These are difficult questions that are not sought to be resolved here.

However, whichever approach is adopted to address the issue, if some animals were accepted as entitled to justice, then their interests could not be ignored in determining the basic structure of society, and the principles of justice. The formulation of the basic liberties of animals might become a key issue, with those basic liberties potentially tailored to the particular moral personality of animals. The implication may be that in a clash between human and animal basic liberties, humans would not necessarily win. Despite the difficulties shown in this article about the position of animals, moral personality is a concept that helps to shed valuable light on the normative basis for human basic liberties. There could even be some potential in the concept as a way to understand what the basic liberties of animals should be.

Granting basic liberties to animals no doubt raises thorny issues about their position in relation to humans both in society and in nature. Would a human duty to respect animals’ basic liberties be restricted to those animals under our direct control, such as farmed animals and companion animals, or extend to those animals in nature that we affect? Alternatively, might there even be a duty to intervene in nature or natural processes to safeguard the basic liberties of animals? Nussbaum observes that, despite the “horrible suffering” inflicted on animals through predation in nature, “[w]e are very ignorant, and if we tried to interfere with predation on a large scale we would very likely cause disaster on a large scale” (p. 248). That is clearly so, and it is also not necessarily the case that we would be obliged to positively protect—as opposed to avoid violating ourselves—the basic liberties of animals. However, these matters, too, are left for another day.

## Data Availability

Data sharing not applicable.
